# One shot ancient character recognition with siamese similarity network

**DOI:** 10.1038/s41598-022-18986-z

**Published:** 2022-09-01

**Authors:** Xuxing Liu, Weize Gao, Rankang Li, Yu Xiong, Xiaoqin Tang, Shanxiong Chen

**Affiliations:** College of Computer and Information Science, Southwest University, Chongqing, 400715 China

**Keywords:** Computer science, Computational science

## Abstract

Ancient character recognition is not only important for the study and understanding of ancient history but also has a profound impact on the inheritance and development of national culture. In order to reduce the study of difficult professional knowledge of ancient characters, and meanwhile overcome the lack of data, class imbalance, diversification of glyphs, and open set recognition problems in ancient characters, we propose a Siamese similarity network based on a similarity learning method to directly learn input similarity and then apply the trained model to establish one shot classification task for recognition. Multi-scale fusion backbone structure and embedded structure are proposed in the network to improve the model's ability to extract features. We also propose the soft similarity contrast loss function for the first time, which ensures the optimization of similar images with higher similarity and different classes of images with greater differences while reducing the over-optimization of back-propagation leading to model overfitting. Specially, we propose a cumulative class prototype based on our network to solve the deviation problem of the mean class prototype and obtain a good class representation. Since new ancient characters can still be found in reality, our model has the ability to reject unknown categories while identifying new ones. A large number of experiments show that our proposed method has achieved high-efficiency discriminative performance and obtained the best performance over the methods of traditional deep learning and other classic one-shot learning.

## Introduction

Ancient characters have far-reaching research value as treasures that record ancient history, economy, culture, and scientific and technological development. After the long-term efforts of paleographers, more and more ancient character materials have been sorted out. However, researchers who use computers to solve ancient character recognition are also discouraged by the lack of relevant domain knowledge. Unfortunately, even for ancient characters researchers with lots of domain knowledge, it is very time-consuming to identify these unmarked ancient characters and even discover new ones. The study of ancient character recognition by computer can not only greatly improve the repetitive behavior of manual processing of character identification, but also efficiently explore the constant pattern of characters in the historical changes using neural networks which can effectively help researchers to conduct further investigation of history and culture.

Different from general handwritten Chinese character recognition^1–3^, ancient character recognition has more difficult problems. Firstly, for example, handwritten Chinese characters or numbers can easily obtain thousands of training data, but it is difficult to obtain a large amount of ancient character data. Secondly, the distribution of the number of characters among ancient characters is extremely unbalanced, and some characters even have only a few sample data. Moreover, the variation of similar characters in ancient characters is large, unlike the differences caused by different writers in Chinese character recognition, and there is more variability in the characters themselves caused by the number of strokes or different shapes. The similarity of ancient characters between different types of characters is extremely disturbing to the recognition accuracy. There are also practical situations where ancient characters are still being excavated and we generalize existing methods to these unfamiliar new classes, which may be expensive due to limited data and extensive retraining. Thus, the existence of various difficulties in ancient characters is also a great challenge for recognition.

To eliminate the dependence on a large amount of data, few-shot learning is becoming a hot spot for researchers in various fields^4–6^, but there is almost no application in ancient character recognition. In particular, the task of using a single sample to recognize the pattern is called one-shot learning^7^, which is to recognize the category matched by the test image in the support set composed of only one picture in each category. In areas where it is difficult to collect a large amount of training data, such as human faces and ancient characters, the method of one-shot learning is very desirable. There are still many problems in ancient character recognition such as the insufficient amount of data and imbalance data, more similar characters of the same kind of variants and similar characters of different classes, and open set recognition to predict unseen class^8,9^.

On the one hand, we hope to develop an effective ancient character similarity matching model, based on which we can develop automatic recognition and retrieval tools to facilitate the work of archaeologists and paleontologists. On the other hand, as part of pattern recognition, visual analysis of images reveals little difference in pattern recognition in similar fields, and it is hoped that our research will not be limited to ancient character recognition but will also be useful for general character or sketch-based related fields.

Overall, this work has three main contributions: First, this paper proposes a Siamese similarity network framework that does not rely on specific domain knowledge to solve the data imbalance, insufficient data, and variant character recognition problems in ancient character recognition. Second, this paper proposes the soft similarity contrast loss function for the first time. It ensures the optimization of similar images with higher similarity and different classes of images with greater differences while reducing the over-optimization of back-propagation leading to model overfitting. This not only improves the recognition accuracy of similar images of different classes, but also makes an important contribution to similarity models and metric learning methods. And we propose a cumulative class prototype to obtain a better class representation. Third, our model can discover new ancient characters and perform ancient character open set recognition, which means it can reject instances in unknown categories.

## Related work

Archaeologists and palaeographers have made long-term unremitting efforts in the study of ancient characters, but their efficiency in solving the recognition and interpretation of unknown characters is relatively poor. In recent years, some researchers have started with computer vision to process and analyze ancient characters^10,11^. Guo et al.^12^ proposed a new multi-level representation that combines Gabor^13^ related low-level representation and sparse self-encoding high-level representation to recognize ancient Oracle and sketch characters^14^. Narang et al.^15^ proposed a joint SIFT^16^ and Gabor features for handwritten ancient Devanagari character recognition. These traditional recognition methods need to manually design domain-adapted features, so the generalization ability of such algorithms and the stability of recognition performance are difficult to guarantee. One-class classification approach^17^ has been applied to determine if the input data is seen class or unseen class which is efficient to find new ancient characters, but it is still hard to distinguish new ones from others.

Currently, deep learning^18^ has reached state-of-the-art performance on various pattern recognition tasks, especially on visual classification^19^ problems. Compared with traditional methods based on rules and manually designed features, deep convolutional networks^20^ have a greater advantage in terms of generalization ability and performance in processing images. Zhao et al.^21^ used feedback from convolutional neural networks to determine an algorithmic model of clustering labeling hyperparameters to improve the recognition rate of ancient handwritten Shui characters. Ghanim et al.^22^ used hierarchical clustering techniques and ranking algorithms to rank cluster members, and finally studied the impact of six different deep convolutional neural networks on Arabic character recognition. However, when these neural network-based algorithms are forced to make predictions on a small amount of available data, they tend to crash due to severe overfitting leading to difficulty in training. Zhang et al.^23^ proposed a triplet network based on deep metric learning, which maps character images to Euclidean space as feature vectors and then uses nearest neighbor classifiers for oracle recognition. Due to its triplet training approach, it is difficult to train to meaningful training samples, which leads to its slow training learning and high computational cost. And the recognition performance is not monitored during the training process thus the generated model has very poor generalization ability.

Thus, it is necessary to propose new one-shot learning methods to be applied in the field of ancient character recognition. Data augmentation^24^ is the most common method used in ancient character recognition, but the extremely small data space leads to a very limited transformation pattern and does not fundamentally solve the overfitting problem. Transfer learning^25^ is also a more common research method, and the performance of pre-trained networks is greatly reduced when the target dataset differs significantly from the source domain dataset. The metric-based approach of one-shot learning is simpler and more efficient, and the data will have different representations based on different tasks. Especially, learning task-based representations can achieve better performance on high-dimensional data. One of the most representative achievements is the method based on the Siamese network proposed by Koch et al.^26^ which rank the similarity between inputs and perform classification recognition. The biggest contribution of the model is to use the ability of the verification model to distinguish the similarity, which is directly used for one-shot recognition and has a good effect on new class recognition. Later, Vinyals et al.^27^ proposed to use a matching network to predict the test set category by learning embedding vectors on the support set using a cosine-based attention mechanism. The model uses segmented sampled mini-batch data to simulate the test task during training, which can reduce the difference between training and testing, thereby improving the generalization performance on the test set. Snell et al.^28^ further explored the relationship between the class embedding vectors in the embedding space, and believed that there is a prototype expression for each category, and then proposed a prototype network. In the article, the class embedding vectors are closely clustered around the class representatives, which is the mean value of the embedding vector of the support set, so the classification problem becomes the category of finding the nearest neighbor of the class prototype representative of the test image, and good results have been achieved. The Siamese structure has been widely used in many fields, such as image recognition^26^, visual tracking^29^, and person re-identification^30^. Drawing on the Siamese network-based one-shot image recognition method proposed by Koch^26^, this paper specifically improves it based on the problems in ancient characters and proposes Siamese similarity network (SSN) for end-to-end one-shot recognition of ancient characters.

Compared with the traditional Siamese network, SSN mainly makes the following improvements : (1) The Siamese backbone in SSN uses a large number of multi-scale feature fusion structures and embedding structures instead of traditional simple convolution to extract image fusion features; (2) Based on the idea of deep metric learning and contrast learning, soft similarity contrast loss (SSCL) is proposed in SSN to train the model so that the similarity of similar ancient characters is higher and the similarity of different ancient characters is lower. The prediction and recognition performance of the model is generalized by the powerful discriminative ability; (3) Inspired by the prototype concept, the trained SSN is used to calculate the cumulative similarity value to obtain the cumulative class representative prototype which is more robust than the original random class prototype and the mean class prototype. The traditional deep network usually utilizes a model with many parameters and then uses a large amount of data to prevent overfitting, while the SSN in this paper can obtain a large number of image pairs from a small amount of training data to train the parameters thus reducing model overfitting.

## Methodology

### General strategy

Methods based on deep metric learning and one-shot learning have achieved very good performance in various fields of pattern recognition. However, the research directly using these methods in the field of ancient character recognition is very rare. In this paper, the proposed method works by proposing a deep metric learning method to learn a good image representation, then directly reusing the features of the network without any retraining, and finally building a one-shot task for nearest neighbor classification. The multi-categorization problem of imbalanced datasets is transformed into a simple validation problem, where the input to the model will be balanced positive and negative sample pairs, and the sampling of balanced positive and negative sample pairs will eliminate the problem of category imbalance even if the data set categories themselves are unbalanced.

**Figure 1:**
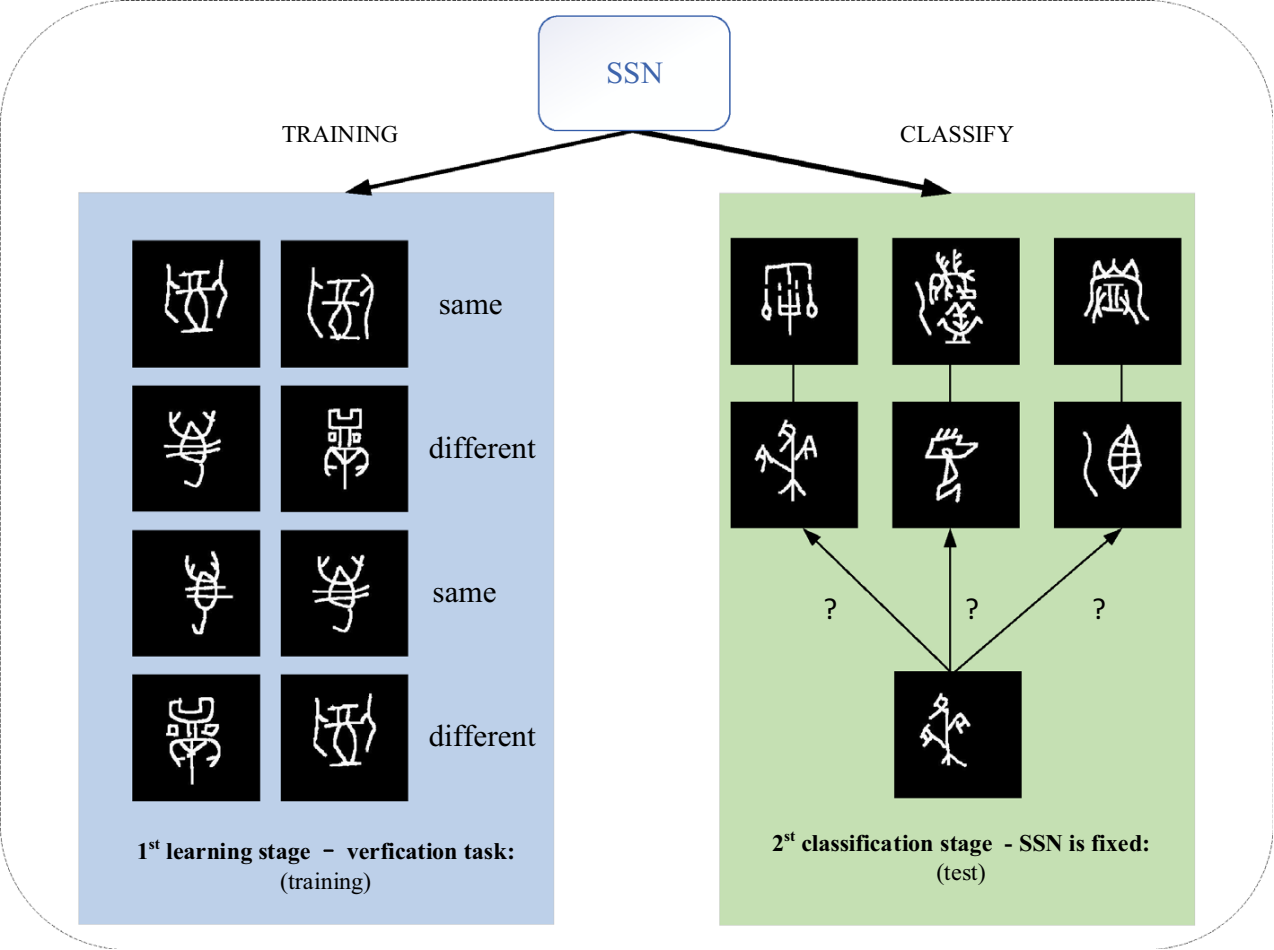
The general strategy. We adopt a two-stage end-to-end recognition framework. In the learning stage, a good representation of the SSN model is obtained through a series of verification tasks, and in the classification stage, the trained model is used to perform nearest neighbor classification.

Figure 1 shows the proposed recognition strategy, which constructs an end-to-end two-stage single-sample recognition framework. In the first learning stage, by inputting a large number of image pairs which can be obtained under the condition of insufficient data for the verification task, we learn a verification model that can distinguish the sample pairs and even give a similarity score, which is the Siamese similarity network (SSN) proposed in this paper. Among them, it is proposed to use a multi-scale fusion network as the backbone of SSN as well as to add embedding structures to obtain more abundant scale information. Subsequently, more accurate fusion distances can be calculated and the distance layer is simply mapped to the similarity probabilities. Finally, a more efficient and accurate gradient value can be obtained by using the SSCL to update the parameter weights. At this stage, our SSN learned the ability to distinguish between similar or different classes of images. In the second classification stage, all parameters of the previously trained SSN are fixed and we directly use them for one-shot classification. We determine the class to which the test image belongs by solving for the highest similarity value of the input image pair. Different from the mean clustering center in the prototype network, we propose to choose the more representative and robust cumulative clustering center as our class prototype. In this stage, the most similar sample classification is completed by the similarity score values output by SSN. If the features learned in SSN are sufficient to confirm the test character classes from some known classes, then after acquiring the ability to match character pair differences under extensive training, our model can also reject instances of unknown classes and find new classes. This is more practical and valuable because in reality new classes are still being discovered.

### Network structure

**Figure 2:**
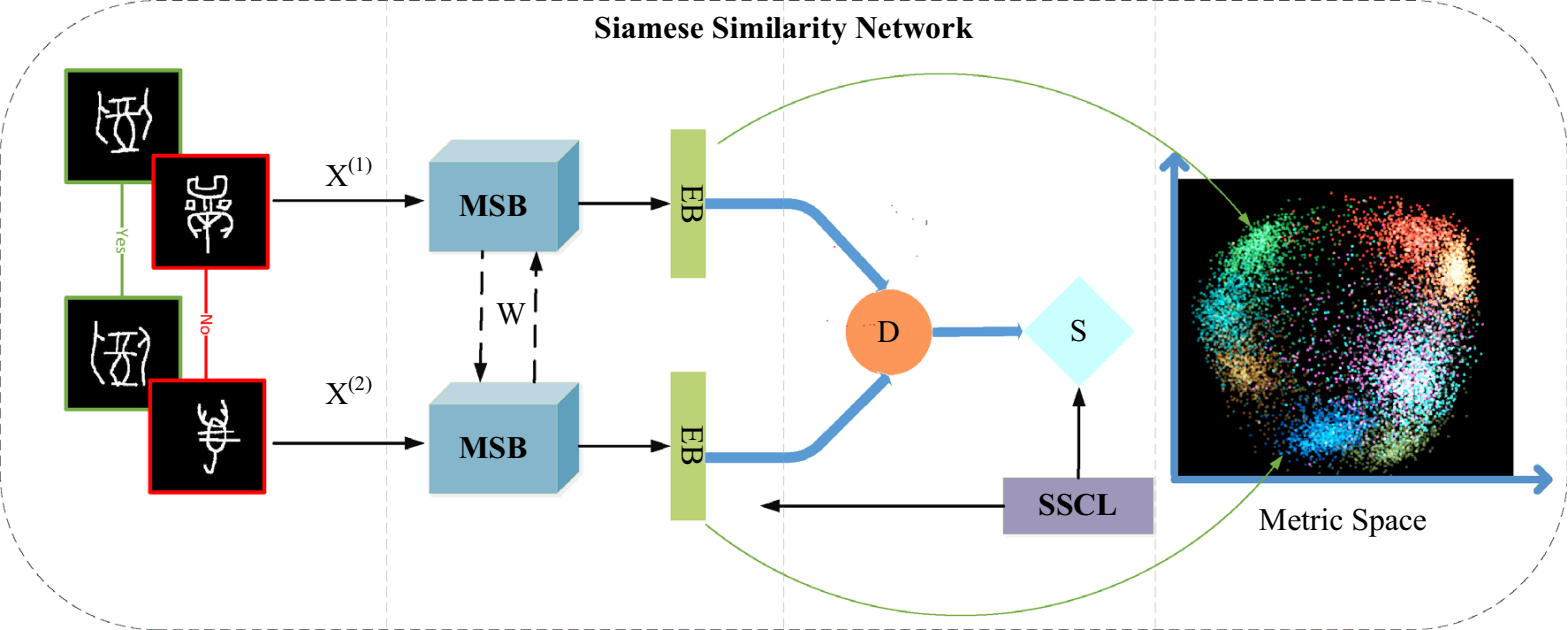
The structure of proposed SSN. It mainly includes multi-scale fusion backbone structure (MSB), embedding structure (EB), fusion distance layer (D), similarity layer (S) and soft similarity contrast loss (SSCL).

Figure 2 illustrates the structure of our SSN. It receives two characters images ${X}^{(1)},{X}^{(2)}$ of the same or different categories. Firstly, the character image features $F({X}^{(1)},F({X}^{(2)})$ are extracted through the processing of Multi-Scale Backbone, and then the corresponding feature embedding representations $E\left(F\left({X}^{\left(1\right)}\right)\right),E(F\left({X}^{\left(2\right)}\right))$ are obtained through a special fusion embedding structure. Accordingly, these two feature vectors are passed through the proposed non-parametric fusion distance layer to obtain the semantic distance values $D({X}^{(1)},{X}^{(2)})$ of the two images, at which time the fusion distance is a weighted sum of the cosine distance and the Euclidean distance, which represents a simultaneous constraint on the distance in terms of value and direction, that is ${D}_{union}=\alpha {D}_{eul}+(1-\alpha ){D}_{cos}$. Since distance and similarity are closely related, images with large distances are less similar to each other and images with small distances are more similar to each other. We use a simple mapping layer containing one node to obtain the final similarity score $S({X}^{\left(1\right)},{X}^{\left(2\right)})$, and apply the softmax function to restrict the similarity score to be between 0 and 1. Our embedding representations show a clear clustering feature in the metric space, which finally means that the higher the similarity of characters with the same category and the lower the similarity of characters with different categories.

#### Multi-scale fusion backbone structure

Due to the simple convolutional layer stack used in the previous Siamese network^26^ or the more traditional classical neural network^31^, we have been aware that these simple convolutional structures have limited non-linear learning capabilities in complex data sets and there are large variations in ancient character. Based on the network structure in Szegedy et al.^32^, we use the MSB module that contains a large number of multi-scale channel fusions as our feature extraction, including a large number of receptive field convolution kernels of various scales and different shapes, which play an important role in capturing multi-scale features and local features. Since increasing the network depth is proven to better extract the target features, our network will use a large number of residuals^33^ to skip the connection structure so that the network can be deepened while reducing the degradation phenomenon. The proposed MSB module uses a large number of different size perceptual fields to obtain multiscale features and rich local features, which are very important for obtaining invariant features in variant characters, thus improving the recognition accuracy of variant characters to a certain extent.

**Figure 3:**
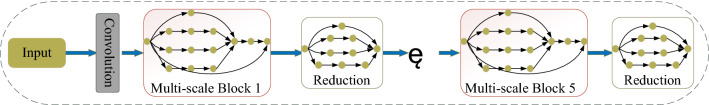
The architecture of MSB module. The input of MSB is the $256\times 256\times 1$ ancient character image. The original image is first obtained by a simple convolution process to obtain the shallow features of the image, and then there are 5 consecutive multi-scale blocks, each of which is followed by a reduction module.

Our MSB mainly consists of five consecutive multi-scale blocks, each of which is followed by a reduction block. Figure 3 briefly illustrates the MSB module. In each multi-scale block, there are multi-scale and multi-shape convolution channels containing 4 receptive field branches of 1*1, 3*3, 5*5, and 7*7, namely C(1), C(3), C(5), C(7). All branches use the same padding which tries to pad evenly left and right and a default stride of one which makes the size of the feature map in each block unchanged. The first channel is 32*1*1 convolution, the second channel is 32*1*1, 32*1*3, and 64*3*1 convolution, and the third channel is 32*1*1, 32*1*5 and 64*5*1 convolution, and the fourth channel is 32*1*1, 32*1*7 and 64*7*1 convolution. The fusion of the multi-scale features of each channel is followed by the 1*1 convolution to normalize the number of channels, and finally the multi-scale feature fusion feature is obtained through residual connection. It can be expressed as:1

In Eq. (1), the double Plus "" represents the concatenation operation and the "" symbol refers to the convolution operation. $R({x}_{concat})$ refers to the concatenation of the feature maps produced in four multi-scale branches. $x$ and $Y$ denote the input and output features of the multi-scale block respectively. The size of the feature map after passing through the multi-scale block does not change, which is subsampled by the reduction block which does convolution and pooling. Our reduction block also contains four branches. All of the branches use the same padding as in the multi-scale block and a stride of two in the only last node of each branch which halves the size of the feature map in the reduction block. The first branch is 3*3 maximum pooling, the second branch is 32*1*1 and 64*3*3 convolution, the third branch is 32*1*1 and 64*5*5 convolution, and the four branches are 32*1*1, 32*3*3 and 64*5*5 convolutions.

#### Embedding structure

**Figure 4:**
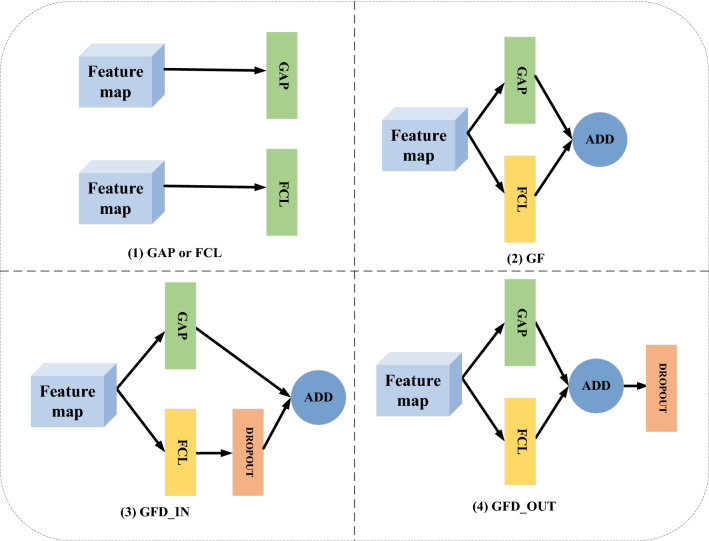
Traditional embedding structure and our residual embedding structure. (1) is the traditional embedding structure and (2) (3) (4) is our proposed residual embedding structure.

Since the traditional embedding structure only uses the Fully Connected Layer (FCL) to vectorize the feature map, the structure often results in severe overfitting due to the need to optimize a large number of parameters, which results in extremely poor generalization performance of this network^34,35,36,37^. Another kind of embedding structure, namely Global average pooling (GAP)^34^, will lose a lot of detailed information due to its rough processing method, and may slow down the convergence speed. Inspired by the idea of residual learning in^33^, we therefore use the residual structure to link the combination of FCL, GAP, and Dropout^35^ as our embedding structure. Our EB structures ensure that more detailed information is added while reducing the number of parameters, which is helpful to improve classification performance and generalization ability. And three EB structures are proposed in Fig. 4, namely the embedding structure with only parallel GAP and FCL (GF), the embedding structure with Dropout in FCL (GFD_IN) and the embedding structure with Dropout in the outermost layer (GFD_OUT). The ADD operation means the eltwise sum. Specially, our FCL is just one dense layer with Relu activation containing the same dimension as GAP layer. Unlike the residual learning of the original feature map in the residual network, our embedding structure adds a richer set of image features learned through the fully connected layer to the vector obtained after global average pooling, using the following equation.

2 $$\begin{array}{c}\left\{\begin{array}{c}Y=R\left(x\right)+GAP\left(x\right) or\\ Y=R\left(x\right)+GAP\left(x\right)+dropout\\ R\left(x\right)=F\left(x\right) or F\left(x\right)+dropout\end{array}\right.\end{array}$$

The symbols of *x* and *Y* denote the input and output of this embedding structure, respectively. $GAP(x)$ denotes the global average pooling of the input to obtain the original embedding representation. $R(x)$ denotes the residual mapping to be learned, and the residual features learned from the fully connected layer are added to the embedding representation via GAP to obtain the most informative and accurate embedding representation. $F(x)$ which means the fully connected layer can be adjusted to represent the same number of channels as the original embedding.

### Learning strategies

#### Soft similarity contrast loss function

In this paper, we design a new loss function based on the traditional contrast loss (CL)^38^, called the soft similarity contrast loss function (SSCL), which is expressed as shown in Eq. (3) below. The proposed loss function can not only implicitly define the similarity measure which is the end-to-end similarity value of the output two characters images but also achieve the goal of metric learning that the similarity of similar characters is high while the similarity of different characters is low. In addition, we consider that contrast loss can over-optimize and thus lead to poor generalization performance. Therefore, our loss function emphasizes that the optimization object is between certain threshold values, otherwise no optimization is performed. It can prevent over-optimization from bringing overfitting, reduce problems such as incorrect optimization, and also speed up the optimization speed.

Specifically, owing to the upper bound of similarity, optimization will be stopped when the similarity value of two images is higher than a certain upper bound. Similarly, there is a lower bound of dissimilarity, and optimization will be stopped when the dissimilarity value of two images is lower than a certain lower bound.

3 $$\begin{array}{c}L\left({x}^{\left(1\right)},{x}^{\left(2\right)}\right)={y}^{\left(1\right)\left(2\right)}\mathrm{max}\left(\left(a-s\left({x}^{\left(1\right)},{x}^{\left(2\right)}\right)\right),0\right)+\left(1-{y}^{\left(1\right)\left(2\right)}\right){\left(s\left({x}^{\left(1\right)},{x}^{\left(2\right)}\right)-b\right)}_{+}\end{array}$$

**Figure 5:**
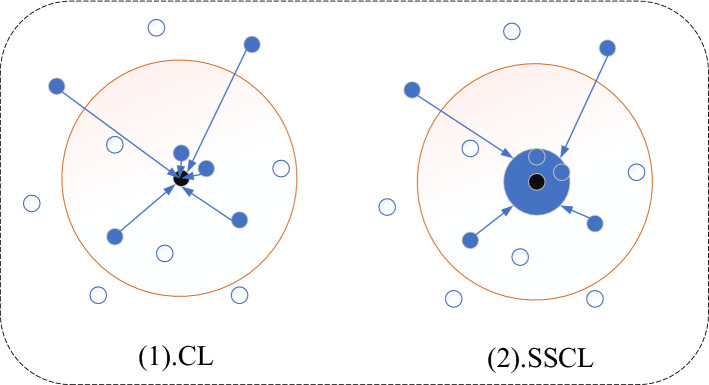
Two loss functions. The blue dots indicate that similar objects need to be as close as possible in the metric space, and the black dots indicate the ideal class center representation. (1) denotes the traditional contrast loss, which requires the objects with high similarity to be brought infinitely close. (2) denotes our proposed soft similarity contrast loss, which stops over-optimization when the high similarity ones are brought closer to a higher threshold to speed up the convergence rate.

${y}^{(1)(2)}$ denotes the labels of two images. if the two images are of the same category, then ${y}^{(1)(2)}=1$, and ${y}^{(1)(2)}=0$ if they are of different categories. The parameters $a$ and $b$ denote the upper bound threshold for reducing over-optimization of similar images and the lower bound threshold for over-optimization of images of different categories, respectively. $s\left({x}^{\left(1\right)},{x}^{\left(2\right)}\right)$ denotes the similarity score value output by SSN. As shown in Fig. 5, CL will continue to optimize when the two objectives are extremely similar while SSCL will optimize up to a certain threshold to stop the optimization, which can avoid the problems caused by over-optimization and speed up the optimization in the meantime. It is obvious that SSCL is faster and more reasonable than CL. The experiments in the supplementary material illustrate these at the end of this paper. The proposed SSCL designed in this paper helps researchers to explore the study of similarity models, and this more direct way of similarity measurement will help to bridge the gap between validation models and classification models.

#### Training strategies

Since our model uses a recognition method that combines the verification model and the nearest neighbor one-shot classification, we have two strategies for monitoring performance during the training process. One is to set a verification task to monitor the generalization performance of the verification model, which requires that the verification model performance can be extended to the recognition model. The other way is to set the one-shot recognition task directly during the verification process to monitor the recognition performance so that the training process is closer to the real recognition scenario to obtain the optimal recognition generalization performance. In practice, we choose both monitoring methods and use the second method as our training termination criterion, which is more consistent with the final real one-shot recognition scenario. And it is proved that the verification model that performs well in one-shot task also performs well in the verification task.

#### Cumulative class prototype

The approach of the mean class prototype (MCP) is proposed in the prototype network^28^, which can represent this class of character images to some extent. When there is a large deviation in a certain class of a certain image, such as the target foreground is small, the background is large, the target is partially obscured or the sample image contains only part of the target, etc., the contribution of such images to the mean class prototype will have a great impact. The method of taking the mean value can easily make the class prototype deviate from the class center and make it difficult to obtain a good class representation, thus having a certain impact on the final recognition effect.

**Figure 6:**
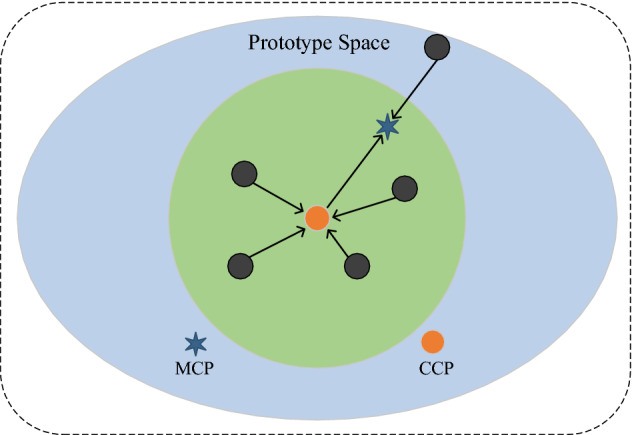
Our CCP. The solid circles represent instances of a particular class of images, the green six-pointed stars represent the mean class prototype, and the orange solid circles represent the cumulative class prototype. The extreme values outside the large circle pull the mean class prototype away from the class center while the cumulative class representatives near the class center have larger similarity values to serve as class representatives.

In this paper, we propose a cumulative class prototype (CCP) based on SSN to solve the deviation problem of this mean class prototype. In the verification task stage, our model hasn’t learned the ability to distinguish between the same or different types of character, and at this stage our model still uses the random class prototypes for model training. In the classification task stage, after our model has learned the ability to distinguish different characters, we will replace the original random class prototype with CCP for one-shot classification. The specific cumulative class prototype is calculated by the formula shown in (4).

4 $$\begin{array}{c}\left\{\begin{array}{c}ACC\left({x}_{j}\right)=\sum\nolimits_{i=1}^{N}S\left({x}_{i},{x}_{j}\right)\\ Proto\left(x\right)=argmax\left\{ACC\left({x}_{1}\right),ACC\left({x}_{2}\right)\dots ACC\left({x}_{N}\right)\right\}\end{array}\right.\end{array}$$

where $x$ denotes a certain category of ancient character images, $i$ and $j$ denote a certain one of such images, respectively. $N$ denotes the total number of images contained in this category. $S$ denotes the similarity score of two images calculated by SSN. $ACC$ means the cumulative similarity score of one image in this category, and finally the image with the maximum value in $ACC$ is taken as our cumulative class prototype $Proto$. As shown in Fig. 6, we found that since there is only one particular sample, which deviates from the more representative clustering center by calculating the mean class prototype. And using our proposed cumulative class prototype, we find that such deviated images we do not achieve a high cumulative similarity score will be eliminated.

### One-shot classification

After learning a verification task consisting of a large number of pairs of ancient character images, our SSN can be directly used in the one-shot classification of ancient characters. There exists a support set S taking N ancient character prototypes composed of one representative image per class, namely N-way one shot, which is denoted as $S=\{\left({x}_{1},{y}_{1}\right),\left({x}_{2},{y}_{2}\right),..,({x}_{N},{y}_{N})\}$. y denotes the labels of such character images. For two images of ancient characters, our SSN will give the corresponding similarity score, which has the powerful distinguishing performance of judging the same class or different one. Therefore, for the category of the test image of ancient characters, the distinguishing performance of SSN is used to find the prototype of the test image with the largest similarity score, so that the category of the test image can be judged for effective classification and recognition.

More importantly, our classification based on one-shot learning is an open-set classification method. In reality, some ancient characters are still being discovered, so an effective open set recognition method is also very important for ancient character recognition. Our model can reject unknown ancient characters and recognize new ones. Experiments show that our method can still deal with the open set problem and get excellent recognition performance.

For unknown character images, which belong to different categories from those in the support set, the obtained similarity score value is relatively low. Therefore, setting a suitable threshold value can reject such unknown images. Once the new class prototypes are identified, our model recognizes instances of these newly discovered classes. Even though more and more class prototypes are added to be recognized, our model still achieves good recognition performance.

## Experiment

### Dataset

We conduct experiments on six datasets: Omniglot, HWAYI, HWOBC, CASIA-AHCDB, OBC306, Bronze-10&Oracle-57.

Omniglot: Omniglot^39^ is a public dataset of handwritten characters for one-shot learning consisting of 1623 categories with a total of 32,460 images. Each category has 20 images. Each of these characters is a 105 × 105 binary image.

HWAYI: The HWAYI dataset^40^ contains a total of 112,031 handwritten ancient Yi characters in 1764 categories (27–96 images per category), and each character image has been normalized to 64 × 64 size.

HWOBC: The handwritten oracle dataset HWOBC^41^ collected a total of 83,245 sample images in 3881 categories. (Each category has 19–24 images).

CASIA-AHCDB: A large-scale Chinese ancient handwritten characters database contains six datasets. We choose AHCDB-style1 of Basic Category^42^ as our experimental dataset which contains annotated character samples of 2,621 categories. It provides 865,940 samples for training and 264,431 samples for testing.

OBC306: OBC306^43^ is a rubbing oracle bone character dataset. It contains a total of 309,551 images covering 306 different classes. The minimum category has only 1 image, and the maximum has 25,898 images.

Bronze-10&Oracle-57: The two datasets were provided by oracle experts from Capital Normal University. Bronze-10 contains a dataset of 10 categories totaling 38 inscriptions (3–4 images per category), and Oracle-57 contains an oracle dataset of 57 categories totaling 1036 images (8–20 images per category).

**Figure 7:**
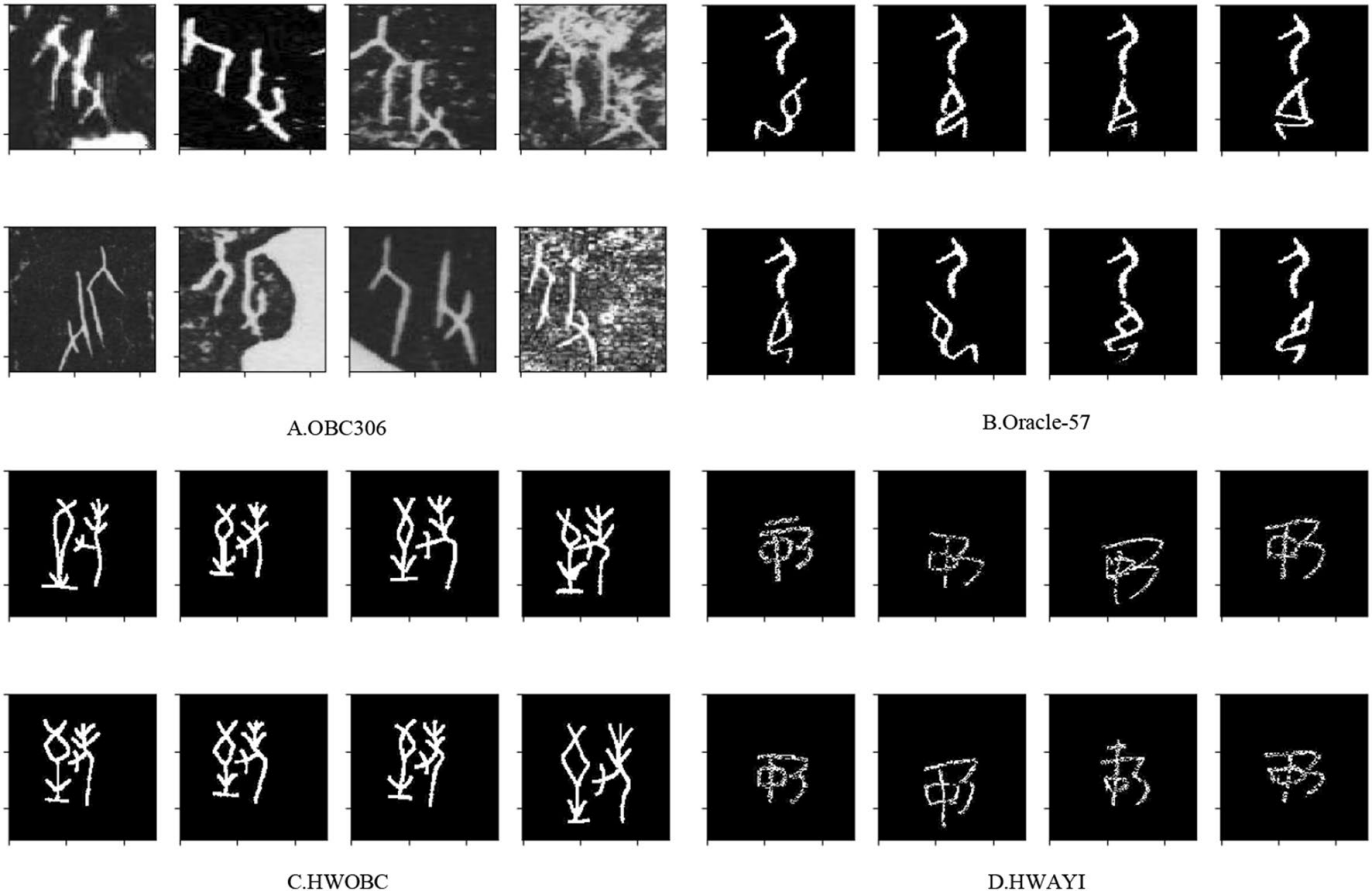
a variety of different images from four different characters in our datasets.

To obtain the best experimental results, we did a series of pre-processing on the initial dataset of ancient character images. Firstly, considering that multi-channel color images do not affect the recognition accuracy, we first convert them into single-channel grayscale images in order to simplify the calculation. Secondly, to facilitate network training, we scaled the images to $224\times 224$ uniformly. Lateral inhibition^44^ is a phenomenon in which adjacent receptors are able to inhibit each other. Since the phenomenon of lateral inhibition produces a stronger visual impact on the form of images with white characters on a black background, we transformed all the pictures of the dataset except OBC306 into the form of white characters on a black background. Finally, we add a black bounding box with a width of 16 pixels to the image becoming a uniform $256\times 256$ scale. Such a border avoids possible boundary effects during the execution of convolution and pooling. As shown in Fig. 7, some instances of our datasets are illustrated.

Using SiameseNet^26^ as a benchmark, we first validate our proposed MSB, EB, SSCL, and CCP on Omniglot. Then we explore the recognition performance, rejection of unknown categories, and open-set classification performance using our SSN on two ancient character datasets, HWAYI and HWOBC. Finally, we conduct comparative experiments on these three datasets with the classic method of one-shot learning and validate the generalization ability of our model on other datasets. The research in this paper is based on the TensorFlow framework and the hardware operating platform uses Nvidia GeForce RTX 2080s GPUs.

### Validation of proposed structures

#### MSB and EB

To efficiently compare other classical feature extraction networks, we used the same hyperparameter settings, optimization algorithms, distance representation, and loss functions as in^26^. The difference only lies in the Siamese backbone and the embedding structures, where the Siamese backbone are the simple CNN structures in the benchmark, vgg16^45^, resnet50^33^, inceptionv3^46^ and our MSB, and the embedding structures are the traditional two structures(FCL, GAP) and our proposed three EB(GF, GFD_IN, GFD_OUT). The final EB setting of 2048 dimensions is the most appropriate in the experiment.

The results of the study are shown in Table 1, and the experiments demonstrate that our MSB significance outperforms other models. Meanwhile, the introduction of a more informative EB obtains better performance than the traditional structure. The accuracy of using the simple convolutional structure model in the benchmark can reach 92% while using the multiscale feature fusion module in this paper can exceed the original paper by 2.39%, and the accuracy after the joint proposed embedding structure reaches 95.72%, and the improvement in the Siamese branch makes our model exceed the original paper by 3.72%, achieving the best results.

**Table 1:** The comparisons of different networks and different embedding structure. Acc means the 20-way one-shot recognition accuracy on Omniglot dataset. The best-performing method is bolded.

Acc (%)	Base	Vgg16	Resnet50	Inceptionv3	MSB (Ours)
FCL	88.72	87.01	93.58	93.81	94.39
GAP	87.09	88.04	93.67	93.96	94.44
GF (Ours)	88.94	88.25	93.91	94.58	94.93
GFD_IN (Ours)	88.93	88.60	93.86	94.47	95.27
GFD_OUT (Ours)	**89.25**	**88.75**	**93.96**	**94.76**	**95.72**

#### SSCL and CCP

To achieve a better data representation to obtain higher similarity scores for similar images and lower similarity scores for dissimilar images of different classes, we propose SSCL to obtain such a good representation. This is also the most essential difference between the baseline and the method in this paper, which introduces the idea of similarity learning to improve the robustness of the model. Considering the evaluation of the final one-shot classification performance, this paper obtains the similarity value based on the trained SSN to obtain the CCP, which is another major innovation of this paper.

Following the parameter settings and the optimal structure of the joint MSB and FGD_OUT from the above experiments, the difference mainly lies in the loss function and the class prototype. The loss function contains the cross-entropy loss function in the benchmark, the CL, and our SSCL. The approach of CL combined with the nearest neighbor classifier (NN) is used for recognition, while all others are tested for one-shot classification performance. The class prototypes used for one-shot recognition are the random class prototype, MCP, and our CCP. The random class prototype refers to a randomly selected class of representative images, while MCP and CCP are both computed by applying the model directly to each class of images after it has acquired some discriminative power through metric learning.

The experimental results are shown in Table 2. The SSCL proposed in this paper obtains better recognition performance than the cross-entropy loss function and CL. Meanwhile, we find that the CCP obtains better performance than the random class prototype and MCP, and the combination of SSCL and CCP even achieves the best performance. So far, our best model has reduced the error rate by 5.81% compared to the benchmark. Thus, we validate the proposed SSN and obtain the state-of-the-art performance.

**Table 2:** Loss functions and class prototypes.

Method	Error
cross entropy	2.73%
CL + NN	2.82%
SSCL + random	2.64%
SSCL + MCP	2.46%
SSCL + CCP	**2.19%**

Significant values are in bold.

### Ancient character recognition

#### Recognition performance

We conduct ablation experiments on three ancient character handwriting datasets to evaluate the efficient classification performance of our proposed method. In this part, SSN uses 60% of the data for training and monitoring, and the remaining 40% for testing one-shot performance. We perform 5-way one-shot and 20-way one-shot recognition tasks with a total of 550 one-shot learning trials per task, from which we calculate the classification accuracy. The dropout rate is set to 0.5 in the experiments and the fusion distance is set to 0.1 to reach the best. To efficiently compare each of our proposed structures, we will use the same hyperparameters, optimizers, and weight initialization as in the benchmark. Then MSB, EB, SSCL, and CCP will be added to this structure in turn.

The experimental results are shown in Table 3. Not only can our ablation experiments demonstrate the important role of each module on SSN, the experimental results also further prove the superiority of our proposed model. From the first row, it can be seen that by using MSB and EB, there is a large improvement in the 5-way and 20-way classification performance. By replacing the distance layer metric with the control variables method, we find that the optimal performance is obtained using a fusion distance. From the second row, it can be seen that the proposed SSCL has a significant effect on the improvement of the model, and reaches state-of-the-art with CCP. In the HWAYI dataset, our best model outperforms the benchmark by 4.43% and 5.64% in 5-way and 20-way, respectively. Similarly, in the HWOBC dataset, our best model outperforms the benchmark by 3.98% and 5.69% in 5-way and 20-way, respectively. Similarly, in the CASIA-AHCDB dataset, our best model outperforms the benchmark by 5.28% and 6.94% in 5-way and 20-way, respectively. Thus, our model has a large improvement over the traditional Siamese model and achieves the performance of state-of-the-art.

**Table 3:** One-shot classification performance of our method on three datasets, the best-performing method is highlighted. “MED” indicates integration of MSB, EB and D in the Fig. 2. “MEDS” indicates integration of MSB, EB, D and SSCL.

Method	Accuracy of one-shot classification (%)							
	Omniglot		HWAYI		HWOBC		CASIA-AHCDB	
	5-way	20-way	5-way	20-way	5-way	20-way	5-way	20-way
base	96.27	92.0	95.32	92.18	95.27	91.81	94.36	91.18
MED(eul)	97.18	94.2	97.89	95.25	98.55	95.27	96.64	94.52
MED(cos)	97.55	94.75	97.56	94.55	98.0	94.36	97.32	95.24
MED(0.1)	98.12	96.81	98.12	95.5	98.75	96.18	98.12	95.45
MEDS + Random	98.18	97.36	99.0	96.91	98.91	96.36	98.32	96.36
MEDS + MCP	99.25	97.54	99.5	97.64	99.0	97.0	98.75	97.55
MEDS + CCP	**99.36**	**97.81**	**99.75**	**97.82**	**99.25**	**97.5**	**99.64**	**98.12**

Significant values are in bold.

#### Rejection performance

By judging the similarity score our model can reject the instances of unknown categories. We choose to conduct rejection experiments on HWOBC with a large number of categories. We randomly select 1552 categories from 3881 categories as unknown categories, and the remaining 60% of categories are used to train and monitor the model. Among the known categories, 1552 categories are randomly selected and 3 untrained images from each category are used to calculate the nearest neighbor similarity score, and the same number of images from each unknown category is selected to calculate the similarity value.

**Figure 8:**
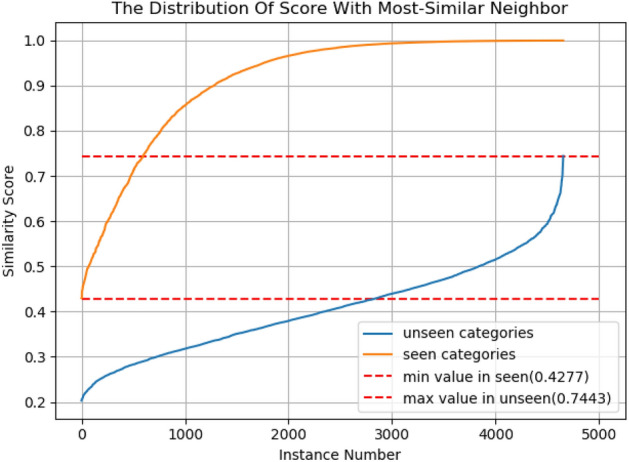
The distribution of similarity score with the nearest neighbour for seen and unseen categories on HWOBC. The vertical axis means the similarity score, the horizontal axis means the instance number. The red dashed line indicates the upper and lower bound thresholds we use to reject instances of the unseen category.

Figure 8 shows a large number of nearest neighbor similarity scores for known and unknown categories. We find that all images in known categories have nearest neighbor similarity scores greater than 0.4277, and all images in unknown categories have nearest neighbor similarity scores less than 0.7443. Therefore, we can set a rejection threshold T in this interval. We denote the accuracy of correctly rejecting the unknown category as ${ACC}_{TN}$ , and the accuracy of correctly receiving the known category as ${ACC}_{TP}$ . We need to maximize the accuracy of these to obtain the best rejection while reducing the number of incorrectly rejected instances. However, due to the overlap of some of the similarity scores, ${ACC}_{TN}$ and ${ACC}_{TP}$ have an opposite relationship. Thus, we choose a suitable threshold T by maximizing the sum of ${ACC}_{TN}$ and ${ACC}_{TP}$. After some estimation and calculation, we obtain an optimal threshold of 0.6002. We also obtain 96.97% for ${ACC}_{TN}$ and 94.50% for ${ACC}_{TP}$. Therefore, our method not only has good recognition performance but also performs efficient rejection for unknown classes of instances, which is important for the study of ancient characters for the discovery of new ones.

Further analysis of rejection performance can be found in Table 4. We randomly select 40% of categories as unknown categories, and the remaining 60% of categories are used to train and monitor the model. Three images from each category are used to calculate the similarity score. Our model can yield a better rejection performance, achieving an accuracy of over 93% for unseen characters and over 92% for seen characters correctly. We can obtain a better rejection threshold while ensuring the best recognition rates for both. In HWOBC, we obtained a threshold of 0.6002 and found that a more optimal threshold was obtained with more categories.

**Table 4:** The character rejection performance of our method on four datasets. “minV”: min similarity value in known categories. “maxV”: max similarity value in unknown categories. “T”: the rejection threshold (the ideal threshold is 0.5).

Datasets	Instance number	minV	maxV	$ACC_{{TN}}$	$ACC_{{TP}}$	T	|T-0.5|
Omniglot	3894	0.4198	0.6832	93.42%	93.79%	0.6188	0.1188
HWAYI	4236	0.3563	0.7628	94.86%	92.18%	0.6147	0.1147
CASIA-AHCDB	6288	0.4244	0.7165	95.35%	94.32%	0.6116	0.1116
HWOBC	9312	0.4277	0.7443	96.97%	94.50%	0.6002	0.1002

#### Open-set recognition performance

Similar to the above experiments, we divide 10% of the HWOBC into a total of 388 categories as unknown new categories, and the remaining 90% is used for SSN training and monitoring the 20-way one-shot recognition performance. To evaluate our open-set recognition performance, we will add 38 categories to the test set in batches until the last 388 unknown categories are added, and obtain the recognition accuracy of the new unknown instance for each batch. Here we explore nearest neighbor classification and random classification as comparative experiments of our method.

**Figure 9:**
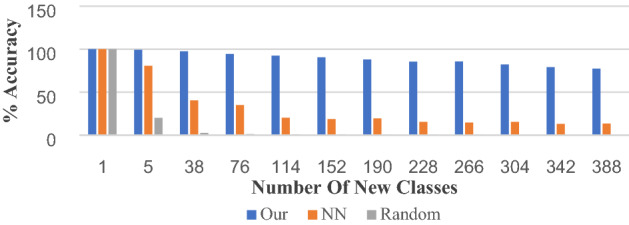
Open set of new class recognition. NN represents the nearest neighbor classifier, the horizontal axis represents a total of 10 batches with the number of classes 1–388, and the vertical axis represents the one-shot recognition rate.

The experimental results are shown in Fig. 9. When we increase the number of new classes of ancient characters to be recognized, we find that our SSN can still maintain a high recognition rate compared with the nearest neighbor and random guess methods, which not only shows that our model can solve the problem of directly recognizing new classes without retraining the model or changing the model structure but also shows that our model performs well when increasing the number of new classes.

### One-shot recognition comparison experiments

To further explore the proposed network, we choose other classic networks in the field of one-shot recognition to conduct comparative experiments. Therefore, in addition to the traditional augmented softmax classification method which uses the main structure of SSN with the last classification layer of 20 nodes, we also choose to test the 20-way one-shot performance of Siamese networks, matching networks, prototype networks, relation networks, and Meta-LSTM in the three datasets mentioned above. Fine-tuning indicates whether the model will be trained using a new category from the test set, which is a common method of fine-tuning for few-shot learning and can yield extra gains.

The above experimental results with several excellent networks show that the proposed SSN achieves better recognition performance than related mainstream methods. Compared with the traditional supervised softmax classification network by augmentation, our method still maintains excellent performance. As shown in Table 5, our model improves about 5.8~6.1% compared to the traditional Siamese network, about 2.3~6.5% compared to the matching network, about 1.5~2.3% compared to the prototype network, about 0.2~0.7% compared to the relation network, and about 3.8~4.2% compared to Meta-LSTM, which reflect the superiority of our proposed one-shot method. Our metric-based one-shot recognition method benefits from a good distance metric. In addition, the method of this paper obtains better recognition results after fine-tuning. In summary, not only in the field of ancient characters our SSN reaches the best classification performance but also in the field of one-shot learning it is an important contribution.

**Table 5:** The comparison experiments. (Except for our benchmark derived from the original paper, the other results are from the reproduced 20-way one shot classification with the best accuracy). “Eul.”: Euclidean distance. “Cos.”: Cosine distance. “Fus.”: Fusion distance of our SSN. “No” means that no test set categories will be used to assist in training the model, while “Yes” means that test set categories will be used to assist in training the model.

Method	Distance metric	Fine-tune	Accuracy (%)		
			Omniglot	HWAYI	HWOBC
augmented + softmax	–	No	96.6	97.5	97.7
SiameseNet^26^ + ConvNet	Eul	No	92.0	92.2	91.8
SiameseNet^26^ + MSB + EB	Eul	No	94.2	95.4	95.8
MatchingNet^27^	Cos	No	93.8	92.7	91.4
MatchingNet^27^	Cos	Yes	94.1	93.5	92.8
MatchingNet FCE^27^	Cos	No	94.5	94.3	93.2
MatchingNet FCE^27^	Cos	Yes	95.4	95.1	95.6
ProtypeNet^28^	Eul	No	96.0	96.5	96.3
RelationNet^47^	–	No	97.6	97.7	97.4
Meta-LSTM^48^	–	No	94.2	93.8	94.1
SSN (Ours)	Eul	No	97.1	97.4	96.6
SSN (Ours)	Cos	No	96.8	96.3	96.9
SSN (Ours)	Fus	No	97.8	97.8	97.5
SSN (Ours)	Fus	Yes	**98.3**	**98.0**	**97.9**

Significant values are in bold.

### Generalization experiments

Studying the transfer performance not only can explore the generalization ability and robustness of our model, but also can make a great contribution to other studies on ancient character recognition with insufficient data. Since the datasets in our experiments are very similar to general ancient character datasets, we use the optimal models obtained on Omniglot, HWAYI, and HWOBC in the above experiments directly or fine-tuned for the other three datasets for all classes of one-shot recognition.

Table 6 shows the experimental results, which indicate that our model has good generalization ability. By directly transferring the models pre-trained on other datasets to these three datasets, our models can obtain better recognition results. In addition, the models obtained by monitoring the three datasets during the training phase with simple fine-tuning can slightly improve the recognition accuracy. Due to the homogeneity of the datasets, we can see that the best model obtained in HWOBC transferred to Oracle-57 and OBC306 can obtain better performance than the other two models. It can be known that our model is insensitive to the categories and focuses more on the variability between characters. And the optimal model obtained on other ancient character datasets can obtain excellent generalization performance without retraining. This is a great encouragement for scholars who study the more limited data of ancient characters recognition.

**Table 6:** Exploring the generalization ability of the model and transfer to other ancient character datasets for recognition accuracy.

Dataset	Fine-tune	Accuracy in our best model (%)		
		Omniglot	HWOBC	HWAYI
Bronze-10	No	87.0	90.5	90.9
	Yes	89.2	91.7	**92.6**
Oracle-57	No	68.8	75.7	72.9
	Yes	71.0	**76.3**	74.3
OBC306	No	45.7	56.8	49.4
	Yes	47.0	**57.6**	51.3

Significant values are in bold.

## Conclusions

In this paper, based on the method of one-shot recognition to analyze ancient characters, we propose a recognition method that uses Siamese similarity network to calculate the similarity of image pairs for one-shot classification. In our approach, MSB and EB are used to obtain more abundant image features to improve the recognition of variant characters, and the proposed SSCL will result in higher similarity scores for similar images and lower scores for different classes of images, and CCP is used to obtain a better representation of image classes. Experiments show that the proposed method achieves the best recognition accuracy than previous methods on these datasets. In addition, our model can reject unknown classes and recognize new classes, and it achieves better recognition accuracy even without retraining, demonstrating the excellent generalization performance of our model.

In future work, we will explore more methods based on deep metric learning to obtain better image representation and choose better multi-scale models to increase the recognition performance of variant characters. In addition, we not only plan to use the proposed model for the discovery of new ancient characters but also apply it to the recognition of ancient characters in more realistic scenes and more recognition problems based on shapes or sketches.

## Supplementary Information

Supplementary Information.

## Data Availability

The datasets used during the current study are available from the corresponding author on reasonable request, and we confirm these data will be public for other studies very soon once our paper is published. Please pay attention to the data resources in the public domain: http://swusmart.cn/getInfo?table=news.
