# Supplementary material for: One shot ancient character recognition with siamese similarity network
Source: Sci Rep. 2022 Sep 1;12:14820. doi: 10.1038/s41598-022-18986-z (PMC9436983; doi:10.1038/s41598-022-18986-z)
Supplement: Supplementary file 1 — Supplementary Information. [file 41598_2022_18986_MOESM1_ESM.docx]

**Supplementary material**

1. CIFAR Experiments

We further validate the effectiveness of the proposed SSCL on the cifar10 dataset in speeding up model convergence and reducing the problem of over-optimization. We use the same best SSN model as in the paper for the validation task. We use SGD with lr = 0.0001 for 150 epochs, weight decay =0.0003, momentum=0.9. Figure A.1 shows the validation error rate of contrast loss (CL) and soft similarity contrast loss (SSCL).


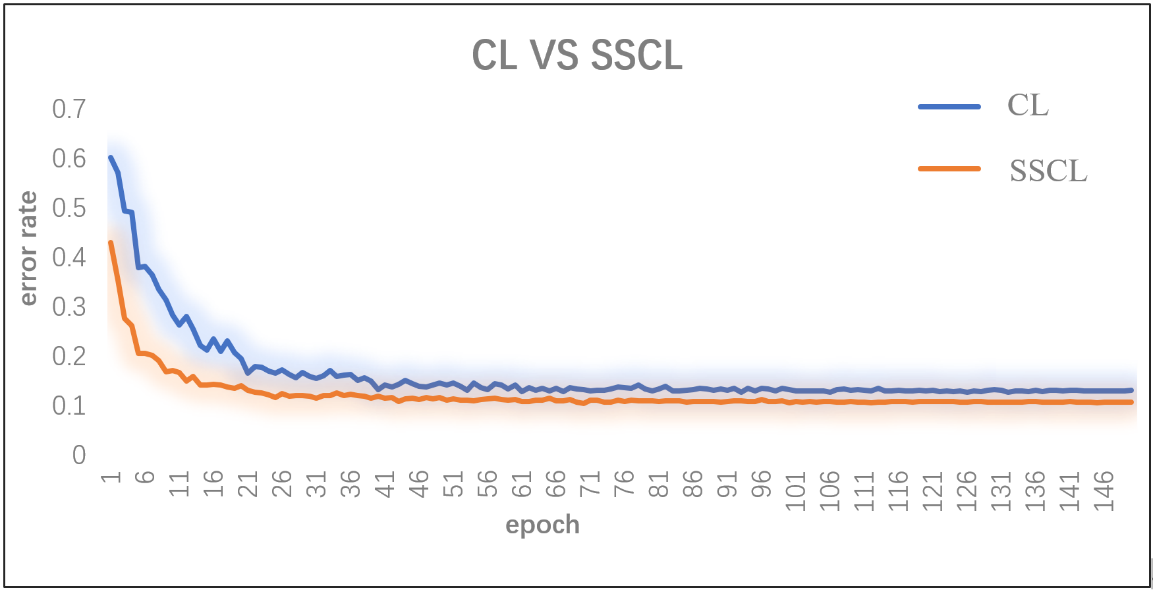


**Figure A.1** CIFAR-10 experiments. Validation error of SSN in verification task of CL and SSCL.

It can be seen in the above figure that the validation error rate of our verification task has been reduced to very low in 6 epochs of training, and the model converges to a stable situation very quickly in the early stage of training, and the accuracy of the final SSCL has been improved compared to the CL. The proposed loss can avoid the problems caused by over-optimization and speed up the optimization at the meantime.
